# A literature review for the introduction of psychiatric simulation to University of Liverpool Medical School

**DOI:** 10.1192/bjo.2021.371

**Published:** 2021-06-18

**Authors:** Alexander Challinor, Declan Hyland

**Affiliations:** 1 School of Medicine at the University of Liverpool, Cheshire and Wirral Partnership NHS Foundation Trust; 2 School of Medicine at the University of Liverpool, Mersey Care NHS Foundation Trust

## Abstract

**Aims:**

The aim of this review is to systematically investigate simulation in psychiatry to enable the evidence based introduction of psychiatry simulation into the undergraduate curriculum at the University of Liverpool.

**Background:**

Transformations in the structure of psychiatric delivery and reductions in funding to mental health care have limited the availability of direct patient clinical experiences for medical students. Experiential learning through simulation can be utilised as a powerful pedagogical tool and provide exposure to a broad range of psychopathology.

Although psychiatric skills and knowledge are gained from the current University of Liverpool undergraduate curriculum, there is no specific well-designed psychiatry simulation.

**Method:**

The author searched MEDLINE, EMBASE and PsycINFO databases for studies that met the inclusion criteria. Search terms included ‘simulation (psychiatry or ‘mental health’). Studies were also searched using snowballing via citation tracking within the databases.

Inclusion criteria comprised studies of an educational intervention that involved simulation. The intervention had to be utilised within the field of psychiatric teaching.

**Result:**

The literature review illustrated the dearth of studies analysing role-playing (RP) and/or simulated patients (SP) in psychiatry with it typically encountered as part of the more general communication skills curriculum. Studies analysing SP and RPs demonstrate how they build on the social context of learning alongside drawing on a range of educational theories, including experiential learning. However, studies show that well-designed simulation training should encompass more facets of learning to be transformative, specifically reflecting upon ones experiences alongside understanding and interpreting this new knowledge, allowing it to guide future actions and change practice.

Studies analysing virtual-reality in psychiatry are limited but demonstrate significant improvements in students’ acquisition of key psychiatric skills and exposure to psychopathology. More studies are needed to evaluate the efficiency and cost-effectiveness of virtual-reality over more traditional methods.

Despite the increase in simulation teaching within psychiatry, and the expansion of innovative simulation approaches in other specialties, there was limited use of novel approaches found within the studies analysing psychiatric simulation. There were studies evaluating novel approaches to psychiatry simulation outside of the undergraduate curriculum.

**Conclusion:**

Whilst there are barriers to overcome in simulation training, these are primarily logistical and are clearly outweighed by the educational gain demonstrated throughout this review. Simulation training in psychiatry has often remained limited to traditional communication-oriented scenarios using RP or SP. A greater emphasis on furthering the advancement and integration of more innovative approaches into psychiatric undergraduate teaching is needed.

